# Integrated Assessment of Shelf Life Evolution in Small Marine Fish from the Northwestern Spanish Coast: Microbial, Chemical, and Sensory Changes During Chilled Storage

**DOI:** 10.3390/foods15132398

**Published:** 2026-07-06

**Authors:** Santiago P. Aubourg, Alicia C. Mondragón, Marcos Trigo, José M. Miranda, Jorge Barros-Velázquez

**Affiliations:** 1 Department of Food Technology, Marine Research Institute (CSIC), c/E. Cabello 6, 36208 Vigo, Spain; mtrigo@iim.csic.es; 2 Department of Analytical Chemistry, Nutrition and Food Science, School of Veterinary Sciences, University of Santiago de Compostela, 27002 Lugo, Spain; alicia.mondragon@usc.es (A.C.M.); josemanuel.miranda@usc.es (J.M.M.); jorge.barros@usc.es (J.B.-V.)

**Keywords:** chilled storage, sardine, horse mackerel, megrim, microbial activity, lipid oxidation, lipid hydrolysis, sensory acceptance, quality deterioration

## Abstract

An integrated study was conducted to evaluate quality loss in small marine fish—sardine (*Sardina pilchardus*), horse mackerel (*Trachurus trachurus*), and megrim (*Lepidorhombus whiffiagonis*)—during a 9-day chilled storage. A progressive increase in microbial counts was observed in all species; aerobic mesophiles exceeded the spoilage limit (7 log CFU·g^−1^) only in horse mackerel by day 9 (7.02 log CFU·g^−1^), while remaining below this threshold in sardine (4.49 log CFU·g^−1^) and megrim (5.42 log CFU·g^−1^). Lipid oxidation showed species-specific behaviour, with sardine and horse mackerel exhibiting higher peroxide values (up to 19.73 and 10.73 meq O_2_·kg^−1^ lipids, respectively) and TBARS formation, whereas megrim showed limited primary and secondary oxidation but a marked increase in tertiary products (ca. six-fold). Lipid hydrolysis increased significantly, with free fatty acids rising by factors of approximately 4.1 (sardine), 13.7 (horse mackerel), and 18.6 (megrim). Similarly, trimethylamine formation increased markedly, with values rising by factors of about 13.1, 19.0, and 51.8, respectively. Sensory evaluation indicated that all species remained acceptable up to day 6 but were unacceptable by day 9, establishing a shelf life of approximately 6 days. Overall, horse mackerel showed the fastest deterioration, highlighting the need for species-specific shelf life assessment strategies.

## 1. Introduction

Fish and other aquatic species provide products of high nutritional value and major economic importance in many countries [1,2]. Aquatic foods can be derived from fresh species through different kinds of technological processes, including both traditional and advanced methods [3,4,5]. In developed countries, chilled aquatic foods dominate the market due to increasing demand for high-quality products, despite potentially long distances between catching or farming sites and consumers [6,7].

However, the quality of wild and farmed aquatic species declines rapidly during chilled storage after death, making them among the most perishable food products [8,9,10]. Deterioration begins immediately after capture or slaughter, and its progression depends directly on storage conditions, particularly icing. The main spoilage mechanisms include microbial activity, autolysis, and lipid oxidation [11,12]. The rate of deterioration depends on factors such as lipid value, species type, size, feeding status at the catching or slaughter time, the level and composition of the microbial load, and storage temperature [13,14]. Accordingly, considerable interest has been paid to the sensory and nutritional modifications occurring in aquatic species during storage in ice.

Small fish (e.g., mackerel, herring, horse mackerel, pouting, megrim, blue whiting, and sardine) form a vital component of marine and freshwater food webs, serving as a primary energy source for larger predators [2,15]. For humans, they represent a nutrient-dense, sustainable, and affordable source of essential micronutrients, omega-3 fatty acids, and proteins [16,17]. Because they are often consumed whole, these fish provide nutrients from the head, bones, and other parts that are typically discarded in larger species. Small fish have long been a staple food in many low- and middle-income countries, representing a cost-effective and nutrient-rich solution to global hunger and malnutrition [18,19]. Smaller fish are generally less vulnerable to overfishing, partly due to their faster reproduction cycles. Notably, larger fish species tend to accumulate higher levels of toxic heavy metals (e.g., mercury, lead) because these elements bioaccumulate over time as the fish grow and consume contaminated organisms [20,21]. Regarding small fish species, previous studies have examined quality loss during chilled storage of sardine [14,22,23], horse mackerel [10,24,25], and megrim [10,26,27], caught in different locations and stored under varying chilling conditions.

The present study focused on three small fish species: sardine (*Sardina pilchardus*), horse mackerel (*Trachurus trachurus*), and megrim (*Lepidorhombus whiffiagonis*). Its objective was to perform an integrated assessment of quality changes occurring during chilled storage of specimens corresponding to such species captured from the Northwestern Spanish coast. To this end, microbial, chemical, and sensory parameters were simultaneously monitored in order to characterise species-specific spoilage pathways and to provide a comparative evaluation of shelf life evolution under standardized storage conditions.

## 2. Materials and Methods

### 2.1. Raw Fish, Chilled Storage, and Sampling Procedure

Fresh sardine (*Sardina pilchardus*), horse mackerel (*Trachurus trachurus*), and megrim (*Lepidorhombus whiffiagonis*) (48 specimens per species) were caught near the Vigo coast (Northwestern Spain) within an ocean area centred approximately on 42°15′ N, 8°55′ W, during 2024 and transported to the laboratory in ice within 10 h. Fish specimens were obtained from commercial catches and were already dead at the time of purchase; therefore, no euthanasia procedures were conducted as part of this study. The specimens showed the following size ranges: 17–22 cm and 145–157 g (sardine), 16–21 cm and 185–230 g (horse mackerel), and 16–21 cm and 165–195 g (megrim).

For each species, twelve specimens were immediately analysed as the initial sample (day 0). Such specimens were distributed into three batches (*n* = 3), each consisting of four specimens, and were analysed independently for microbial, chemical, and sensory quality.

The remaining specimens were distributed into three batches (36 specimens per batch) and immediately stored in ice at a fish-to-ice ratio of 1:1 (*w*/*w*). Fish were not headed or gutted and were kept in a refrigerated room at 4 °C. Throughout storage, boxes allowing drainage of melted ice were employed, and ice was replenished as necessary to maintain the 1:1 ratio. Sampling was carried out on days 3, 6, and 9. At each sampling time, twelve specimens (three batches of four specimens; *n* = 3) were analysed for microbial, chemical, and sensory parameters, following the same procedure as for day 0.

### 2.2. Determination of Microbial Counts

Microbial enumeration was performed on 10 g portions of fish muscle aseptically excised and homogenised with 90 mL of 0.1% peptone water (Merck, Darmstadt, Germany). Homogenisation was carried out in sterile stomacher bags (AES, Combourg, France) according to previously described procedures [26,28,29]. Homogenates were serially diluted in 0.1% peptone water.

*Enterobacteriaceae* were enumerated on Violet Red Bile Agar (VRBA; Merck) after incubation at 37 °C for 24 h. Aerobic mesophilic bacteria were determined on Plate Count Agar (PCA; Oxoid, London, UK) after incubation at 30 °C for 48 h, and psychrotrophic microorganisms were quantified on PCA following incubation at 7 °C for 7 days. All analyses were carried out in triplicate, with results being expressed as log CFU·g^−1^ of muscle.

### 2.3. Lipid Oxidation Determination

Lipid extraction from fish tissue was performed in agreement with the Bligh and Dyer [30] method. Initially, a chloroform/methanol/water mixture (1.0:2.0:0.8, *v*/*v*/*v*) was employed. Subsequently, chloroform and water were added to achieve a final ratio of 2.0:2.0:1.8 (chloroform/methanol/water, *v*/*v*/*v*), resulting in phase separation. The lower phase, corresponding to the lipid fraction, was collected. Lipid extracts were stored at −40 °C under a nitrogen atmosphere until analysis. Lipid value was determined following the method of Herbes and Allen [31], and results were expressed as g lipids·kg^−1^ of wet tissue.

Conjugated diene (CD) value was determined spectrophotometrically at 233 nm [32] in the lipid extract. Results were calculated as CD = A × V·w^−1^, where A is the absorbance and V and w correspond to the volume (mL) and lipid weight (mg) of the analysed aliquot.

Conjugated triene (CT) value was evaluated at 268 nm [32] and calculated using CT = A × V·w^−1^.

Peroxide value (PV) was determined spectrophotometrically at 520 nm following peroxide reduction with ferric thiocyanate [33]. Results were expressed as meq active oxygen·kg^−1^ of lipids.

The thiobarbituric acid index (TBA-i) was assessed in agreement with Vyncke [34], based on the formation of thiobarbituric acid reactive substances (TBARSs), determined at 532 nm and quantified using a 1,1,3,3-tetraethoxypropane standard curve. Results were calculated as mg malondialdehyde·kg^−1^ of fish muscle.

Fluorescent compounds resulting from interactions between oxidised lipids and protein components were analysed using fluorescence spectroscopy (LS 45 Fluorimeter; PerkinElmer, Spain). Measurements were performed at excitation/emission wavelengths of 393/463 nm and 327/415 nm in the aqueous phase obtained after lipid extraction [35]. Relative fluorescence (RF) was determined as RF = F/Fst, where F is the sample fluorescence and Fst corresponds to a quinine sulphate standard (1 µg·mL^−1^ in 0.05 M H_2_SO_4_). The fluorescence ratio (FR) was then calculated as FR = RF_393_/_463_/RF_327_/_415_.

### 2.4. Lipid Hydrolysis Determination

Free fatty acid (FFA) value was assessed spectrophotometrically at 715 nm in lipid extracts using the procedure proposed by Lowry and Tinsley [36], based on the development of a cupric acetate–pyridine complex. Toluene was used instead of benzene as the organic solvent. Calibration was performed using an oleic acid standard solution (705.3 mg in 25 mL toluene) over a range of 0.5–15.0 μmol (R^2^ = 0.9998). Results were calculated as g FFA·kg^−1^ of lipids.

### 2.5. Determination of Trimethylamine (TMA)

Trimethylamine nitrogen (TMA-N) content was assessed spectrophotometrically at 410 nm using the picrate method in agreement with the method proposed by Tozawa et al. [37]. A 5% trichloroacetic acid extract of fish muscle was prepared (10 g in 25 mL). Calibration was performed using trimethylammonium chloride standards (0.962 mg N·250 mL^−1^) within a range of 1–40 μg of TMA-N (R^2^ = 0.9993). Results were calculated as mg TMA-N·kg^−1^ of fish muscle.

### 2.6. Sensory Evaluation

Sensory analysis was conducted by a trained panel of five experienced assessors, following the guidelines for fresh and chilled fish described in Council Regulation [38] and employed in the previous procedure [29]. All assessors were informed about the nature of the study and provided their written informed consent prior to participation. According to institutional guidelines, formal ethical approval was not required for sensory evaluation studies involving trained panellists and non-invasive assessment of food products.

Samples were prepared in a dedicated room and evaluated under controlled ventilation and lighting conditions. All assessors had demonstrated experience in the sensory evaluation of fresh and chilled fish and underwent specific training sessions based on established freshness grading guidelines prior to the study to ensure consistency and reliability of the evaluations. Four quality categories were used: extra (E), good (A), fair (B), and unacceptable (C). The evaluation included the following descriptors: skin, eyes, gills, and external odour. The detailed scoring rubric corresponding to the E/A/B/C grading system, including the specific evaluation criteria for each sensory descriptor, is provided in Table 1 [29].

Prior to the study, panel members were trained using fresh and chilled specimens of the same species. At each sampling time, fish were presented anonymously (“blind” evaluation), and assessors recorded their scores individually.

### 2.7. Statistical Analysis

After verifying normality (Kolmogorov–Smirnov test) and homogeneity of variances (Bartlett’s test), data were analysed using parametric methods. One-way ANOVA was applied to assess the effect of storage time (*p* < 0.05); Fisher’s least significant difference (LSD) test for mean comparisons was employed. PASW Statistics 18 (SPSS Inc., Chicago, IL, USA) was used for statistical analysis.

Correlation analyses between storage time and microbial or chemical indices were performed using Pearson’s correlation test. Linear models are presented unless otherwise specified (quadratic or logarithmic fitting).

## 3. Results and Discussion

### 3.1. Evaluation of Microbial Development in Chilled Fish

Microbial counts are widely used as key indicators of hygienic quality and spoilage progression in fresh fish, reflecting both initial contamination levels and bacterial growth during storage.

A progressive increase in *Enterobacteriaceae* counts was observed during chilled storage in all three species—sardine (r = 0.90), horse mackerel (r = 0.93), and megrim (r = 0.92)—with the data best described by a quadratic model (Table 2). However, the magnitude and timing of this increase differed markedly among species. Compared with initial (day 0) values, horse mackerel showed a significant (*p* < 0.05) increase from day 6 onward, reaching 4.28 log CFU·g^−1^ by day 9. Megrim exhibited a more moderate but still significant increase (*p* < 0.05) at days 6 and 9, whereas sardine remained comparatively stable, with no significant (*p* > 0.05) changes throughout storage. These results indicate that sardine muscle showed the greatest resistance to *Enterobacteriaceae* proliferation under chilled conditions, followed by megrim, while horse mackerel was the most susceptible species in terms of hygienic quality.

Aerobic mesophilic counts also increased significantly (*p* < 0.05) with storage time in sardine (r = 0.94), horse mackerel (r = 0.93), and megrim (r = 0.95) (Table 2). However, clear interspecies differences were evident. Horse mackerel exhibited the most pronounced microbial growth, with significant increases (*p* < 0.05) from day 6 and levels exceeding the commonly accepted spoilage threshold of 7.0 log CFU·g^−1^ by day 9. In contrast, sardine and megrim showed slower increases. Sardine displayed significant changes (*p* < 0.05) as early as day 3, but final values remained well below the spoilage limit (4.49 log CFU·g^−1^ at day 9). Similarly, megrim reached only 5.42 log CFU·g^−1^ at the end of storage. These findings point to greater resistance to aerobic spoilage in sardine and megrim compared with horse mackerel, with sardine showing the most favourable profile overall.

Psychrotrophic microorganisms followed a similar increasing trend in all species, showing strong correlations with storage time (r = 0.92 for sardine, r = 0.92 for horse mackerel, and r = 0.95 for megrim) (Table 2). Sardine exhibited significant increases (*p* < 0.05) during the 3–9-day period, whereas horse mackerel and megrim showed significant changes (*p* < 0.05) mainly between days 6 and 9. Final psychrotrophic counts exceeded 7.0 log CFU·g^−1^ in all species by day 9; however, megrim surpassed this threshold already at day 6. This earlier increase suggests that, despite its relatively good performance for aerobic mesophiles, megrim was less resistant to psychrotrophic microorganisms, whereas sardine and horse mackerel showed better control of cold-tolerant bacterial growth during intermediate storage. Notably, psychrotrophic bacterial counts exceeding approximately 7 log CFU·g^−1^ are generally considered indicative of advanced spoilage and are associated with unacceptable sensory quality.

It has been indicated that, in living fish, the immune system prevents bacterial proliferation [39]; however, post-mortem collapse allows microorganisms to invade the muscle during chilled storage [40]. The present study establishes clear quantitative relationships between storage time and microbial growth for key microbiological indicators and highlights substantial interspecies differences in spoilage resistance. Aerobic mesophiles, the main spoilage indicator, did not reach unacceptable levels after 6 days of storage in any species, and threshold values were not reached in sardine or megrim even by day 9. In terms of hygienic quality, *Enterobacteriaceae* counts remained below critical levels during early storage in all species and throughout the entire period in sardine and up to day 6 in megrim. Overall, sardine showed the highest resistance to microbial spoilage, followed by megrim, whereas horse mackerel exhibited the fastest deterioration and the shortest shelf life.

The greater resistance of sardine muscle to microbial spoilage during chilled storage can largely be attributed to species-specific biochemical and physiological characteristics. Sardines are fatty pelagic fish rich in long-chain polyunsaturated fatty acids (LC-PUFAs), such as eicosapentaenoic (EPA) and docosahexaenoic (DHA) acids, whose hydrolysis and oxidation products have been reported to exhibit antimicrobial activity against Gram-negative spoilage and hygiene-related bacteria [3,41,42]. In addition, Pedrosa-Menabrito and Regenstein [43] and Ruiz-Capillas et al. [44] indicated that the rapid post-mortem autolysis typical of small pelagic species facilitates the early release of FFAs and low-molecular-weight peptides with inhibitory effects on bacterial growth. These mechanisms are consistent with the limited increase in aerobic mesophiles and *Enterobacteriaceae* observed in sardine throughout storage. Furthermore, pelagic fish generally harbour lower microbial loads on skin and gills than demersal species due to reduced exposure to sediment-associated microbiota [45], supporting the superior microbiological stability observed for sardine.

In contrast, horse mackerel exhibited the most pronounced microbial growth, particularly for aerobic mesophiles. This behaviour can be attributed to its muscle composition and ecological characteristics. Horse mackerel muscle has high water activity and elevated levels of non-protein nitrogen compounds, which favour microbial proliferation during chilled storage [8,41]. Moreover, this species inhabits pelagic–neritic environments and carries a diverse indigenous microbiota, including psychrotolerant spoilage bacteria such as *Pseudomonas* and *Shewanella*, which are capable of rapid growth at refrigeration temperatures [46,47]. These features are consistent with the early attainment of spoilage levels observed in this species.

Megrim, a lean demersal species, displayed intermediate microbial behaviour. Its low lipid content limits the formation of antimicrobial lipid-derived compounds, facilitating the growth of psychrotrophic microorganisms [10,41], while its compact muscle structure and lower metabolic activity appear to delay aerobic mesophilic spoilage. In addition, demersal fish are strongly influenced by sediment-associated cold-adapted microbiota, which represent an important source of psychrotrophic spoilage organisms [47,48].

From a regulatory perspective, the microbial counts observed are consistent with European microbiological criteria for fresh fishery products. According to Regulation (EC) No 2073/2005 [49], *Enterobacteriaceae* are considered hygiene indicators, whereas aerobic counts are commonly used as spoilage indicators. According to these guidelines, *Enterobacteriaceae* levels below 3 log CFU·g^−1^ and aerobic mesophilic counts below 7 log CFU·g^−1^ are generally considered acceptable during shelf life [41,49]. In this context, sardine and megrim remained within acceptable limits for most of the storage period, whereas horse mackerel exceeded the spoilage threshold for aerobic mesophiles at day 9, indicating the end of its shelf life. These findings highlight the relevance of species-specific evaluation, as current regulations do not explicitly account for differences in spoilage kinetics among species.

Microbial growth during chilled storage is driven by interactions between indigenous microbiota, muscle composition, and post-mortem changes. Spoilage is mainly associated with psychrotrophic Gram-negative bacteria such as *Pseudomonas* and *Shewanella*, which are well adapted to low temperatures and dominate under refrigeration [46,47]. Their proliferation explains the increase observed in aerobic mesophilic and psychrotrophic populations, consistent with their recognised role as primary spoilage organisms [8,11]. After death, the collapse of immune defences and tissue integrity facilitates microbial migration into the muscle, accelerating spoilage [39,40].

Differences in microbial development may also reflect variability in initial contamination and environmental origin, as fish microbiota is strongly influenced by habitat conditions [45,48]. Microbial metabolism leads to the formation of volatile compounds responsible for off-odours, linking microbial growth to sensory rejection [11,41]. In this context, aerobic counts approaching 7 log CFU·g^−1^ are commonly associated with unacceptable quality [41,49]. Finally, interactions with endogenous processes may modulate spoilage, as lipid-derived compounds can exert inhibitory effects, whereas the availability of nitrogenous substrates promotes bacterial growth [3,8,41,42]. Overall, microbial spoilage reflects a balance between microbial ecology, substrate availability, and post-mortem changes, which is in agreement with the progressive increases and inter-sample differences observed for the studied microbial groups during chilled storage.

Overall, the shelf life observed in this study is consistent with, and in some cases longer than, that reported in previous studies on chilled fish. In sardine, aerobic mesophilic counts have generally remained well below the widely accepted spoilage limit of 7 log CFU·g^−1^ even after 9 days of refrigeration, exceeding the typical 5–7-day shelf life reported for small pelagic species stored under similar conditions [41,42]. A progressive increase in both aerobic and *Enterobacteriaceae* counts with storage time was observed in specimens caught off the Moroccan Atlantic coast near Agadir, reaching values in the range of 6–7 log CFU·g^−1^ by day 9 for both microbial groups [50]. Similarly, sardine obtained 5–20 km off the Casablanca–Rabat coast and stored for 15 days showed aerobic and psychrotrophic counts within the ranges of 5.3–7.8 and 5.3–7.9 log CFU, respectively, at spoilage levels [22]. In sardine from the Antalya coast (Turkey), aerobic counts remained below 2 log CFU·g^−1^ during a 0–6-day iced storage period [14]. Sardine caught on the Northwestern Spanish coast and stored in ice for up to 22 days reached 7.0 log CFU·g^−1^ for both aerobic and psychrotrophic bacteria by day 19, while values of approximately 5.0 log CFU·g^−1^ were observed at day 8 [51]. Likewise, sardines from the Marmara Sea showed a progressive increase in microbial counts during 9 days of chilled storage, reaching approximately 6.0 log CFU·g^−1^ (aerobes), 5.4 log CFU·g^−1^ (psychrotrophs), and 5.8 log CFU·g^−1^ (*Enterobacteriaceae*) by day 9 [52].

Horse mackerel has consistently shown faster microbial deterioration, with spoilage limits typically reached or exceeded by day 9, in agreement with reported shelf lives of approximately 6–8 days under chilled storage conditions [8,46]. In specimens from the Portuguese coast, aerobic, *Enterobacteriaceae*, and psychrotrophic counts exceeded 7 log CFU·g^−1^ after 10 days of storage, although values remained below this level up to day 8 [53]. A gradual increase in aerobic counts was also observed over a 12-day period, reaching 6.5 log CFU·g^−1^, followed by a slight decrease during extended storage (15–19 days) [29].

Megrim exhibited intermediate behaviour, with aerobic mesophilic counts remaining below spoilage thresholds throughout storage, consistent with reported shelf lives of up to 7–10 days for lean demersal species [10,41]. Megrim specimens obtained at the Grand Sole Bank and stored on ice on board for 14–20 days showed aerobic, psychrotrophic, and *Enterobacteriaceae* counts within the ranges of approximately 3.7–4.4, 5.3–5.9, and 0.7–1.5 log CFU·g^−1^, respectively [26]. In another study, fish captured in the North Atlantic and stored on board for varying periods (3–14 days), followed by up to 11 days of onshore chilled storage, remained acceptable only when onboard storage did not exceed 3 days; longer prior storage resulted in microbial counts exceeding acceptable limits (i.e., above 7.0 log CFU·g^−1^) during the onshore period [27].

### 3.2. Evolution of Lipid Oxidation in Chilled Fish

Lipid oxidation is one of the main deterioration pathways in fish, involving the formation of primary and secondary lipid oxidation compounds. Additionally, the interaction between such oxidised compounds and nucleophilic-type molecules (i.e., protein-like compounds) present in the fish muscle leads to the formation of fluorescent compounds (i.e., tertiary lipid oxidation compounds) that significantly affect quality and shelf life. Initial lipid contents of the white muscle of the three species were 18.57 ± 5.31, 10.22 ± 0.80, and 5.41 ± 0.63 g·kg^−1^ wet muscle for sardine, horse mackerel, and megrim, respectively.

The evolution of lipid oxidation in the muscle of the three studied fish species was evaluated at different stages of this deterioration process. A progressive increase in CDs with storage time was observed in sardine (r = 0.95, quadratic fitting) and megrim (r = 0.92, quadratic fitting), but no clear trend was detected in horse mackerel (Table 3). Compared with initial values, higher average CD levels were recorded at the end of the study for all species; however, statistically significant differences (*p* < 0.05) were only found in sardine muscle.

No significant changes (*p* > 0.05) in CT values were observed in any species during the first 6 days of storage (Table 3). Nevertheless, all species exhibited a significant increase (*p* < 0.05) in CT content by the end of the study compared with initial values. A meaningful correlation with storage time was only observed for horse mackerel (r = 0.93, quadratic fitting).

A marked formation of peroxides (*p* < 0.05) during storage was proved in sardine (r = 0.92, quadratic fitting) and horse mackerel (r = 0.94) (Table 3). In sardine, peroxide values exceeded the 10–15 range by the end of the experiment, which is often considered the acceptability limit for chilled fish and indicative of the onset of rancidity and limited acceptability [54,55]. On the contrary, no significant effect (*p* > 0.05) of chilled storage on PV was observed in megrim, with all values remaining within the narrow range of 1.41–1.71, which can be considered very low for chilled fish species [54,55].

**Figure 1:**
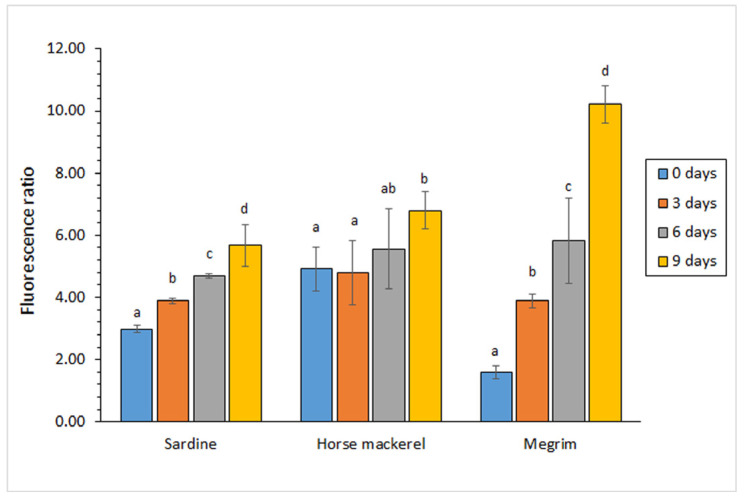
Determination of the fluorescent compound formation (fluorescence ratio) in the fish muscle subjected to chilling storage. Mean values ± standard deviations of three replicates (*n* = 3). For each fish species, values accompanied by different letters denote significant (*p* < 0.05) differences with storage time.

The formation of TBARSs showed a remarkable increase with storage time in sardine (r = 0.95, quadratic fitting) and horse mackerel (r = 0.95, quadratic fitting) (Table 3). Compared with initial values, a significant increase (*p* < 0.05) was already observed at day 3 in both species. In megrim, however, a varying pattern was evident; thus, an initial increase at day 3 was followed by a decrease over the 6–9-day period. The observed decrease in TBARS values in megrim at advanced storage stages can be explained by the rapid transformation of secondary oxidation products into tertiary oxidation compounds. In this species, the marked increase in fluorescent compounds suggests an intense interaction between aldehydic lipid oxidation products (such as malondialdehyde) and nucleophilic groups of proteins and other constituents. As a result, free TBARSs may decrease their content due to their participation in the formation of fluorescent tertiary products, indicating a progression of oxidation rather than a reduction in oxidative damage. This interpretation is consistent with the pronounced fluorescence increase observed in megrim during storage (Figure 1). Notably, all TBARS values in chilled megrim remained below 0.65, which can be considered relatively low [54,55]. TBARS values exceeding approximately 5–8 mg malondialdehyde·kg^−1^ muscle are typically associated with perceptible rancidity and reduced consumer acceptability.

A progressive increase (*p* < 0.05) of fluorescent compound formation was detected in all species as a result of chilled storage, including sardine (r = 0.95), horse mackerel (r = 0.95, quadratic fitting), and megrim (r = 0.95, quadratic fitting) (Figure 1). Compared with initial values, significant increases (*p* < 0.05) were already detected at day 3 in sardine and megrim, with further significant increases at each subsequent sampling time. Fluorescent compound formation was particularly pronounced in megrim, reaching levels approximately six times higher than the initial value by the end of the study. In contrast, horse mackerel exhibited relatively low formation, with significant changes (*p* < 0.05) detected only at the final stage of storage. This behaviour is consistent with previous studies reporting limited primary oxidation development and relatively low peroxide values in horse mackerel during chilled storage [24], as well as a gradual and moderate accumulation of secondary oxidation products such as TBARSs [53], particularly at advanced storage stages.

Lipid oxidation is a complex process including the formation of a wide variety of compounds (i.e., primary, secondary, and tertiary), most of which are unstable and susceptible to breakdown into smaller molecules. Due to their high reactivity and electrophilic nature, these compounds tend to degrade or interact with food constituents containing nucleophilic groups (i.e., –NH_2_ and –SH, corresponding to lysine and methionine compounds), leading to remarkable nutritional losses [56,57,58]. As a consequence, a substantial accumulation of tertiary oxidation products, such as fluorescent compounds, is observed [35,59,60]. Therefore, the measurement of a single degradative class of compounds does not always provide an accurate determination of fish quality decrease. Therefore, the assessment of several lipid oxidation indices is found necessary to obtain a definite knowledge of the oxidative state of the lipid matter. In the current study, CT, PV (except in megrim), TBARS, and FR values proved to be useful indicators of lipid oxidation, whereas CD values did not provide an accurate measure of oxidation degree.

Previous research has evaluated various lipid oxidation indices in these fish species during chilled storage, sometimes focusing on a single parameter and, in other cases, providing a more comprehensive assessment across different oxidation stages. In sardine, Nunes et al. [61] reported a progressive increase in TBA values in specimens captured near Lisbon (Portugal), reaching approximately 15 mg·kg^−1^ (September samples) and 8 mg·kg^−1^ (February samples) after 7 days. Sardine from the Northwestern Spanish coast stored in ice for 8 days showed a progressive increase in CD content from 1.2 to 2.6 [62]. In the same study, a great increase in TBARS value was observed during the first day, followed by a plateau, while fluorescent compounds increased markedly during the subsequent storage period [62]. A later study [63] reported strong development of peroxides and TBARSs during the first 8 days of storage, followed by stabilisation, whereas fluorescent compound presence increased sharply after 12 days. Sardines from the Marmara Sea also showed progressive increases in peroxides and TBARSs up to 9 days [52], reaching approximately 14.9 meq active oxygen·kg^−1^ and 21.5 mg MDA·kg^−1^, respectively. More recently, Janči et al. [23] observed a rapid increase in TBARS value from 0.3 to 8 mg MDA·kg^−1^ during the first 3 days, followed by a slower increase up to approximately 10 mg MDA·kg^−1^ by day 7.

In horse mackerel, Smith et al. [24] reported low peroxide formation (2.8 meq active oxygen·kg^−1^ after 6 days). Da Silva et al. [53] reported an increase in TBARS content from 1.05 to 1.56 mg MDA·kg^−1^ between days 2 and 10. During 19 days of chilled storage, no changes in CD content were detected, but TBARS presence increased progressively, and fluorescent compounds showed a sharp rise at later stages (9–19 days). Similarly, Losada et al. [64] reported increasing TBA values over 15 days, although all values remained below 1.0, while fluorescent compound content increased sharply after day 8. Low PVs were also reported for Danish samples after 96 h [65]. Panguila et al. [25] observed significant secondary oxidation in samples from Angola and Portugal, reaching approximately 11.5 and 7.4 mg MDA·kg^−1^, respectively, after 13 days.

In agreement with the present results, low development of primary and secondary lipid oxidation has been indicated in megrim. Miranda et al. [66] observed peroxide and TBARS values within the ranges of 0.5–2.5 meq active oxygen·kg^−1^ and 0.2–0.4 mg MDA·kg^−1^, respectively, during 14 days of chilled storage of specimens from the Northwestern Spanish Atlantic coast.

### 3.3. Determination of Lipid Hydrolysis in Chilled Fish

FFA formation is a well-recognised indicator of lipid hydrolysis development, resulting from both endogenous enzymatic activity and microbial metabolism during chilling storage of marine species.

**Figure 2:**
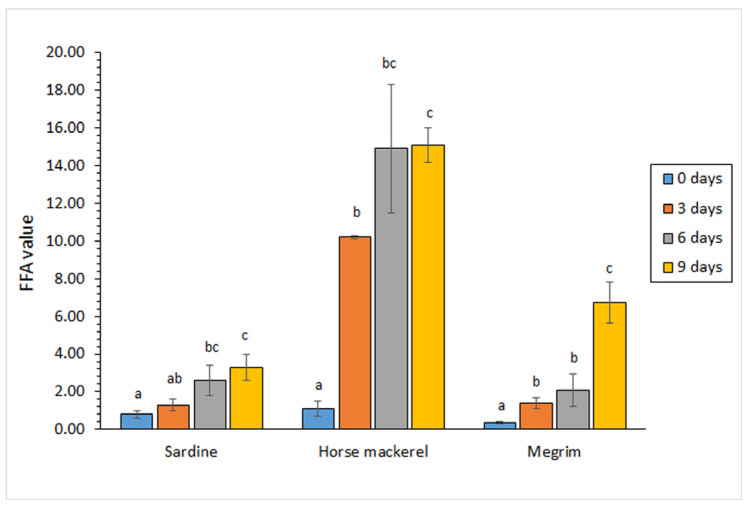
Determination of the free fatty acid (FFA) value (g·kg^−1^ lipids) in the fish muscle subjected to chilling storage. Mean values ± standard deviations of three replicates (*n* = 3). For each fish species, values accompanied by different letters denote significant (*p* < 0.05) differences with storage time.

A significant (*p* < 0.05) and progressive increase in FFA content was detected in all fish species with storage time (Figure 2). This increase was more pronounced in horse mackerel (r = 0.94, logarithmic fitting) and megrim (r = 0.93, quadratic fitting) than in sardine (r = 0.94). Thus, comparison of FFA contents at days 9 and 0 yielded ratio values of approximately 13.7 and 18.6 for horse mackerel and megrim, respectively, whereas a lower ratio (ca. 4.1) was found for sardine.

FFA formation during chilled storage has been described as the result of both endogenous enzymatic activity and microbial activity [3,9]. During the initial stages, prior to the end of the microbial lag phase, FFAs are mainly the result of endogenous lipase and phospholipase activity. As storage progresses, microbial activity becomes increasingly important due to bacterial catabolic processes [9,13]. According to the present results, the lag phase in horse mackerel and megrim appeared to be shorter than in sardine, which explains the greater accumulation of FFAs in the lipid fraction of these species. Although no universally accepted regulatory limit exists, FFA contents above approximately 20–30 g·kg^−1^ lipids are generally associated with advanced lipid hydrolysis and quality deterioration in chilled fish. This value range was not attained in any of the species studied.

While the formation of FFAs itself does not lead to nutritional losses, its assessment is deemed important when considering the development of quality loss [9,13]. Thus, it has been proved that the accumulation of FFAs is to some extent related to lipid oxidation enhancement and to texture deterioration by interaction with proteins. Thus, a pro-oxidant effect of FFAs on lipid matter has been proposed and explained on the basis of a catalytic effect of the carboxyl group on the formation of free radicals by the decomposition of hydroperoxides [63,64].

The present findings are consistent with previous studies reporting FFA formation in these fish species during chilled storage. In sardine, a sharp increase in FFAs was observed during storage for up to 8 days [62], with values rising from approximately 1.1 to 15.9 g·100 g^−1^ of lipids. A progressive increase was also reported over a 22-day icing period [63], with values of approximately 0.4, 0.9, and 1.7 g·100 g^−1^ of lipids at days 8, 19, and 22, respectively.

In horse mackerel, a gradual increase in FFA value was detected during chilled storage for up to 19 days [67], with values rising from approximately 1.4 g·100 g^−1^ of lipids initially to around 2.8 and 5.3 g·100 g^−1^ at days 8 and 19, respectively. In another study, no FFA formation was detected for the first 5 days of storage, followed by a marked increase between days 8 and 19, reaching values of approximately 6 and 25 g·100 g^−1^ of lipids [63]. Similarly, an increase from 0.87 to 1.55 g·100 g^−1^ of lipids was reported after 96 h of chilled storage in specimens from the Danish coast [65].

A marked accumulation of FFAs was also reported in megrim. In specimens captured in the Grand Sole Bank and stored on board under chilled conditions, values of 5.0, 15.9, and 18.8 g·100 g^−1^ of lipids were observed after 14, 16, and 20 days, respectively [26]. Likewise, megrim captured off the Northwestern Spanish Atlantic coast showed a substantial increase in FFAs during 14 days of storage, rising from an initial value of 10.4 g·kg^−1^ lipids to 62.8 g·kg^−1^ lipids at the end of the study [66].

### 3.4. Determination of TMA in Chilled Fish

TMA is a widely accepted indicator of freshness loss in fish; it is mainly produced through microbial degradation of trimethylamine oxide during chilling storage, partly by endogenous enzymatic activity, but predominantly as a result of microbial metabolism.

**Figure 3:**
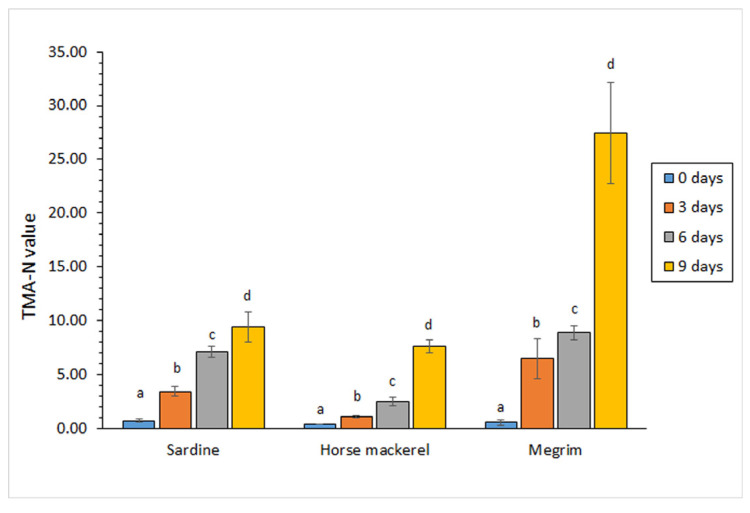
Determination of the trimethylamine-nitrogen (TMA-N) value (g·kg^−1^ muscle) in the fish muscle subjected to chilling storage. Mean values ± standard deviations of three replicates (*n* = 3). For each fish species, values accompanied by different letters denote significant (*p* < 0.05) differences with storage time.

A relevant increase (*p* < 0.05) in trimethylamine-nitrogen (TMA-N) values was observed in all fish samples during storage (Figure 3). Notably, the values recorded at each sampling point were significantly higher (*p* < 0.05) than those obtained at the previous one for all species. Strong correlations with storage time were found for sardine (r = 0.95), horse mackerel (r = 0.94, quadratic fitting), and megrim (r = 0.93, quadratic fitting). Among the species studied, megrim showed the highest TMA-N formation, whereas horse mackerel exhibited the lowest. In this regard, a ratio of approximately 51.8 between day 9 and day 0 values was observed in megrim. Nevertheless, the corresponding ratios for sardine and horse mackerel were also considerable, reaching approximately 13.1 and 19.0, respectively [14,55].

TMA-N value has been widely recognised as a reliable indicator of quality loss during refrigerated storage of fish, due to its strong association with freshness deterioration [11,55]. In general, TMA-N values above approximately 10–15 mg·100 g^−1^ of muscle are commonly associated with loss of freshness and are considered indicative of unacceptable quality for human consumption.

Previous studies have indicated substantial TMA formation in the present fish species during chilled storage. In sardine, specimens caught off the Moroccan Atlantic coast near Agadir exhibited a progressive increase during 18 days of storage, reaching approximately 2.5, 4.8, and 17.5 mg TMA-N·100 g^−1^ of muscle at days 6, 9, and 18, respectively [50]. Similarly, sardine from the Portuguese coast near Lisbon showed a gradual increase during the first 12 days, reaching about 4 mg TMA-N·100 g^−1^ of muscle, followed by a sharp rise during days 14–19, exceeding 13 mg TMA-N·100 g^−1^ of muscle [61]. Sardine captured off the Antalya coast (Turkey) and stored in ice for up to 6 days also showed a progressive increase, reaching approximately 1.6 mg TMA-N·100 g^−1^ of muscle at the end of the experiment [14]. In specimens obtained from the Northwestern Spanish coast, TMA-N values remained below 2 mg TMA-N·100 g^−1^ of muscle during the first 12 days, followed by a marked increase to approximately 11 mg TMA-N·100 g^−1^ of muscle during the 15–22-day period [51]. Sardine from the Marmara Sea showed values of approximately 2.5, 3.4, and 4.2 mg TMA-N·100 g^−1^ of muscle after 1, 5, and 9 days of chilled storage, respectively [52].

In horse mackerel, specimens from the Southwestern England coast stored for up to 12.5 days exhibited a progressive increase in TMA-N, with values of approximately 1.4, 1.8, and 3.4 mg TMA-N·100 g^−1^ of muscle at days 0, 7.5, and 12.5, respectively [24]. No TMA formation was detected during the first 6 days of chilled storage in another study [67], but a sharp increase was observed during days 9–19, reaching about 3.3 and 14.4 mg TMA-N·100 g^−1^ of muscle at days 9 and 19, respectively. Similarly, a slight increase was observed during the initial 0–5-day period in specimens from the Northwestern Spanish coast, followed by a marked increase during days 8–19, reaching approximately 22.5 mg TMA-N·100 g^−1^ of muscle at the end of the study [29].

Regarding megrim, Pastoriza et al. [27] reported TMA formation in specimens obtained from trawlers operating in the North Atlantic. In that study, fish stored on board for 3 days and subsequently for 11 days on shore reached a TMA-N value of 4.0 mg·100 g^−1^ of muscle, whereas fish stored for 8 days on board followed by 11 days on shore reached a value of 45 mg·100 g^−1^ of muscle.

### 3.5. Evolution of Sensory Acceptance in Chilled Fish

Sensory evaluation provides an overall assessment of fish quality by integrating visual, olfactory, and textural attributes, and is considered a key criterion for determining consumer acceptability.

Initially, sardine and horse mackerel were rated as extra quality (E) for all evaluated descriptors, whereas megrim was graded as quality A by the end of the experiment; all species were classified in category C, which corresponds to an unacceptable product and unfit for human consumption (Table 4). Based on these results, a shelf life of approximately 6 days can be established for all species under the present conditions. Among the evaluated descriptors, external odour proved to be the most limiting factor in all cases. Additionally, eye appearance was a key determinant of quality in sardine and megrim, while skin appearance was particularly important in sardine.

A general decrease in sensory quality during chilled storage has also been indicated in previous research on these species. In sardine, specimens obtained from the Moroccan Atlantic coast near Agadir showed a shelf life of 9 days during an 18-day storage period [50]. Similarly, sardine from the Rabat–Casablanca area exhibited a shelf life of 9.5 days during 15 days of storage [22]. On the contrary, a shorter shelf life of 5 days was reported for sardine captured on the Northwestern Spanish coast, where fish were considered unacceptable by day 8 based on external odour, eye appearance, and flesh odour [51]. Sardine from the Marmara Sea stored under chilled conditions for up to 9 days were judged unacceptable at day 9 according to the Quality Index Method, corresponding to a shelf life of 7 days [52].

For horse mackerel, a decrease in sensory acceptance was reported during 12.5 days of chilled storage, with acceptability maintained up to approximately 6.5 days [24]. A progressive decline in quality was also described for specimens from the Portuguese coast during a 10-day storage period, leading to a shelf life of approximately 6 days [53]. Horse mackerel from the Northwestern Spanish coast exhibited a longer shelf life of up to 12 days during a 19-day storage period [67]. In another study, specimens stored in ice showed a shelf life of 5 days and were considered unacceptable by day 8, with external odour, gill condition, and flesh odour identified as limiting descriptors [29]. A decrease in fishy and rancid odour was also reported after 96 h of chilled storage in specimens from the Danish coast [54]. Furthermore, a comparative study on horse mackerel from Angola and Portugal during 13 days of chilled storage indicated that samples became unacceptable by day 7 according to the Quality Index Method [25].

In the case of megrim, specimens captured in the Grand Sole Bank and stored on ice on board showed a shelf life of 16 days, becoming unacceptable by day 20, with external odour and gill appearance identified as limiting descriptors [26]. In another study, megrim from the North Atlantic stored on board for 3, 8, 12, or 14 days and subsequently stored for up to 11 days on shore were considered acceptable only when onshore storage did not exceed 3 days; longer storage periods resulted in unacceptable quality according to the applied quality index [27].

## 4. Conclusions

A progressive decline in microbial, chemical, and sensory quality was observed in all species throughout chilled storage. However, the evolution of spoilage mechanisms showed clear species-specific differences. Microbiological analysis indicated that sardine and megrim generally remained within acceptable limits under the studied conditions, whereas horse mackerel reached spoilage thresholds at later storage stages. Lipid oxidation was more pronounced in sardine and horse mackerel at the primary and secondary levels, while megrim showed a higher accumulation of tertiary oxidation products. Lipid hydrolysis and TMA formation increased markedly in all species, with particularly high values observed in horse mackerel (FFA) and megrim (TMA-N). Despite these differences, all species exhibited a comparable sensory shelf life of approximately 6 days under the present experimental conditions.

These findings underline the importance of considering species-specific characteristics when evaluating fish quality and shelf life. However, the present study has some limitations that should be acknowledged. Only three fish species from a single geographical area were analysed, and storage was performed under a single set of chilled conditions. In addition, factors such as seasonal variability, capture methods, and pre-storage handling conditions, which may influence spoilage dynamics, were not specifically addressed.

Future research should therefore focus on extending this integrative approach to a wider range of species, environmental conditions, and storage systems. In particular, the evaluation of innovative preservation strategies (e.g., natural antioxidants, antimicrobial agents, and advanced chilling technologies) under different real distribution scenarios would provide further insight into improving fish quality and shelf life.

Overall, the results provide a comprehensive and comparative perspective on quality deterioration in small marine fish and contribute to a better understanding of the complex interplay between microbial activity, lipid degradation, and sensory perception. These findings may serve as a useful basis for improving quality assessment and preservation strategies, although further studies are needed to confirm their applicability under broader conditions.

## Figures and Tables

**Table 1:** Scale employed for evaluating the freshness degree of the present chilled species.

Descriptor	Highest Quality (E)	Good Quality (A)	Fair Quality (B)	Unacceptable (C)
Skin	Very intense pigmentation; transparent mucus	Milky mucus; insignificant pigmentation losses	Slightly greyish mucus; pigmentation without shine	Widely opaque mucus; important pigmentation losses
Eyes	Convex; transparent cornea; bright and black pupil	Convex and slightly sunken; slightly opalescent cornea; black and cloudy pupil	Flat; opalescent cornea; opaque pupil	Concave and milky cornea; Internal organs blurred
External odour	Sharply seaweedy and shellfish smell	Weakly seaweedy and shellfish smell	Incipiently putrid and rancid	Putrid and rancid
Gills	Brightly red; without odour; lamina perfectly separated	Rose coloured; without odour; lamina adhered in groups	Slightly pale; incipient fishy odour; lamina adhered in groups	Grey-yellowish colour; intense ammonia odour; lamina totally adhered

**Table 2:** Assessment * of microbial parameters (log CFU·g^−1^ muscle) in the fish muscle subjected to chilling storage **.

Fish Species	Microbial Group	Chilling Time (Days)			
		0	3	6	9
Sardine	*Enterobacteriaceae*	1.10 ± 0.28 a	1.17 ± 0.15 a	1.23 ± 0.24 a	1.93 ± 0.85 a
	Aerobe mesophiles	2.89 ± 0.11 a	3.34 ± 0.16 b	3.77 ± 0.18 b	4.49 ± 0.13 c
	Psychrotrophs	3.86 ± 0.30 a	5.55 ± 0.65 b	5.89 ± 0.13 b	7.81 ± 0.16 c
Horse mackerel	*Enterobacteriaceae*	1.00 ± 0.00 a	1.23 ± 0.40 a	3.03 ± 0.41 b	4.28 ± 0.33 c
	Aerobe mesophiles	2.82 ± 0.24 a	3.53 ± 0.35 a	5.83 ± 0.31 b	7.02 ± 0.25 c
	Psychrotrophs	3.86 ± 0.41 a	4.14 ± 0.20 a	6.26 ± 0.44 b	7.41 ± 0.36 c
Megrim	*Enterobacteriaceae*	1.10 ± 0.17 a	1.64 ± 0.73 ab	1.83 ± 0.42 b	3.77 ± 0.28 c
	Aerobe mesophiles	3.63 ± 0.06 a	4.05 ± 0.58 ab	4.75 ± 0.64 bc	5.42 ± 0.10 c
	Psychrotrophs	5.64 ± 0.25 a	6.39 ± 0.47 ab	7.09 ± 0.69 bc	7.81 ± 0.23 c

* Mean values ± standard deviations of three replicates (*n* = 3). ** For each fish species and for each microbial group, values followed by different letters indicate significant (*p* < 0.05) differences with storage time.

**Table 3:** Determination * of the lipid oxidation evolution in the fish muscle subjected to chilling storage **.

Fish Species	Oxidation Index ***	Chilling Time (Days)			
		0	3	6	9
Sardine	CD	0.60 ± 0.05 a	0.63 ± 0.05 a	0.64 ± 0.06 a	0.84 ± 0.02 b
	CT	0.044 ± 0.001 a	0.042 ± 0.010 a	0.045 ± 0.006 a	0.085 ± 0.019 b
	PV	0.65 ± 0.19 a	2.71 ± 0.37 b	5.05 ± 0.49 c	19.73 ± 2.37 d
	TBA-i	0.42 ±0.03 a	0.88 ± 0.20 b	1.42 ± 0.87 b	2.80 ± 0.38 c
Horse mackerel	CD	0.95 ± 0.01 a	0.99 ± 0.08 a	0.94 ± 0.21 a	1.11 ± 0.07 a
	CT	0.049 ± 0.006 a	0.052 ± 0.001 a	0.062 ± 0.022 ab	0.100 ± 0.033 b
	PV	0.78 ± 0.03 a	3.86 ± 0.52 b	8.76 ± 1.41 c	10.73 ± 2.88 c
	TBA-i	0.10 ±0.05 a	0.22 ± 0.01 b	0.71 ± 0.22 c	1.49 ± 0.40 d
Megrim	CD	0.54 ± 0.04 a	0.59 ± 0.02 a	0.60 ± 0.04 a	0.65 ± 0.18 a
	CT	0.050 ± 0.007 a	0.042 ± 0.003 a	0.047 ± 0.010 a	0.067 ± 0.004 b
	PV	1.71 ± 0.35 a	1.73 ± 0.74 a	1.41 ± 0.58 a	1.62 ± 0.04 a
	TBA-i	0.17 ± 0.05 a	0.62 ± 0.04 d	0.45 ± 0.01 c	0.30 ± 0.02 b

* Mean values ± standard deviations of three replicates (*n* = 3). ** For each fish species and for each oxidation index values followed by different letters indicate significant (*p* < 0.05) differences with storage time. *** Abbreviations and units: PV (peroxide value; milliequivalents active oxygen·kg^−1^ lipids), TBA-i (thiobarbituric acid index; mg malondialdehyde·kg^−1^ muscle), and CD (conjugate dienes) and CT (conjugated trienes) both expressed as indicated in the experimental section.

**Table 4:** Evolution of the sensory acceptance * of fish muscle subjected to chilling storage.

Fish Species	Descriptor	Chilling Time (Days)			
		**0**	**3**	**6**	**9**
Sardine	Skin	E	A	B	C
	Eyes	E	A	B	C
	External odour	E	B	B	C
	Gills	E	A	A	B
Horse mackerel	Skin	E	A	A	B
	Eyes	E	A	B	B
	External odour	E	A	B	C
	Gills	E	A	A	B
Megrim	Skin	A	A	B	B
	Eyes	A	A	B	C
	External odour	A	A	B	C
	Gills	A	A	B	B

* Freshness categories: E (excellent), A (good), B (fair), and C (unacceptable), as indicated in Table 1.

## Data Availability

The original contributions presented in the study are included in the article; further inquiries can be directed to the corresponding author.
